# Root Cause Analysis of Increased Referral Rates in a Sub-district Hospital, Tamil Nadu: A Quality Improvement Initiative

**DOI:** 10.7759/cureus.67470

**Published:** 2024-08-22

**Authors:** Stalin R, Charumathi B

**Affiliations:** 1 Community Medicine, Saveetha Medical College and Hospital, Saveetha Institute of Medical and Technical Sciences, Saveetha University, Chennai, IND

**Keywords:** pareto chart, plan-do-study-act cycle, quality improvement, referral rate, root-cause-analysis

## Abstract

Background

Sub-district hospitals in Tamil Nadu are critical in providing essential healthcare services, but they face significant challenges that can lead to increased patient referrals to higher-level facilities. High referral rates can overburden tertiary care centers, delay specialized treatment, and affect patient outcomes. This study aims to identify the root causes of increased referral rates in a sub-district hospital and implement targeted interventions to reduce unnecessary referrals.

Methods

A descriptive study was conducted at Sriperumbudur sub-district hospital in Tamil Nadu from May to August 2023. The study utilized a root cause analysis (RCA) approach, incorporating qualitative data from brainstorming sessions with healthcare providers and administrative staff, and quantitative data from hospital records on referral rates. A fishbone (Ishikawa) diagram was employed to map causal factors, and Pareto and bar charts were used to analyze and present referral trends. Interventions were implemented using the Plan-Do-Study-Act (PDSA) cycle.

Results

The analysis identified several key factors contributing to high referral rates, including inadequate diagnostic services, insufficient staffing, and lack of essential resources such as CT scans and blood components. Following targeted interventions, referral rates decreased significantly from 101 cases in May-June 2023 to 52 cases in July-August 2023 highlighting a reduction of over 48%. The most notable reductions were seen in referrals for road traffic accidents with head injury (38.7%) reduction, chronic kidney disease (CKD)/hypertension (HT)/diabetes mellitus (DM) (46.2%) reduction, and crush injuries (45.5%) reduction.

Conclusions

The RCA revealed systemic issues that were contributing to increased referral rates at the sub-district hospital. Implementing targeted interventions based on the RCA findings led to a significant reduction in referrals, improving patient care at the local level and alleviating the burden on tertiary care centers. This study underscores the importance of continuous quality improvement initiatives in strengthening healthcare delivery at the sub-district level.

## Introduction

Root cause analysis (RCA) is a systematic process used in the medical field to identify the underlying causes of adverse events and near misses, aiming to enhance patient safety and prevent future incidents. RCA involves detailed data collection, identifying causal factors, and using techniques such as fishbone diagrams to determine fundamental causes. By focusing on systemic issues rather than individual blame, RCA promotes continuous improvement and fosters a culture of transparency and learning. Its implementation leads to robust healthcare processes and significant improvements in patient safety [1].

Sub-district hospitals play a crucial role in providing comprehensive secondary health care and referral services to the population they serve. These hospitals, located below the district level and above the block level (CHC, community health center), act as first referral units (FRUs) for the Tehsil/Taluk/block population. Sub-district hospitals provide essential healthcare services to rural and semi-urban populations, bridging the gap between primary health centers and higher-level district or tertiary hospitals. They offer a range of medical services, including general medicine, maternal and child health, minor surgical procedures, emergency care, and basic diagnostic services. Equipped with outpatient and inpatient facilities, these hospitals play a critical role in managing common illnesses, conducting routine health check-ups, and implementing public health programs [2]. Despite their vital function, sub-district hospitals often face challenges such as limited resources, inadequate staffing, and infrastructure constraints, which can affect the quality and scope of care they provide.

The referral system in healthcare involves sending patients from facilities with fewer resources to those with more resources and specialists. The objectives of referrals include providing priority care, categorizing patients, preventing complications, and providing specialist care. Referral rates from a sub-district hospital can detrimentally impact patient care by causing delays in specialized treatment, overburdening higher-level facilities, increasing healthcare costs, fragmenting patient care continuity, fostering patient dissatisfaction due to extended wait times, highlighting inefficiencies in resource utilization, and potentially demoralizing hospital staff, who may feel constrained in delivering comprehensive care locally. These challenges underscore the need for strengthening sub-district hospitals through improved resource allocation, enhanced medical training, and better integration of primary and secondary healthcare services to minimize unnecessary referrals and ensure timely, effective patient management within the local healthcare system [3].

This study aims to conduct a comprehensive RCA of the factors contributing to the increased referral rates in a sub-district hospital in Tamil Nadu, with the ultimate goal of informing targeted interventions to strengthen healthcare services at the sub-district level, and thereby reducing the burden on the district headquarters hospital.

## Materials and methods

This study employed a descriptive approach to conduct a comprehensive RCA aimed at understanding the factors contributing to increased referral rates from a sub-district hospital in Tamil Nadu. The choice of a descriptive study design was deliberate, as it allows for an in-depth exploration of the various causal factors influencing the phenomenon. This approach facilitated a detailed collection and analysis of both qualitative and quantitative data, offering a holistic view of the challenges faced by the healthcare facility.

The study was conducted at Sriperumbudur sub-district hospital in Tamil Nadu, a healthcare facility that serves a diverse population encompassing both rural and semi-urban communities. The hospital provides essential healthcare services, including general medicine, maternal and child health, minor surgical procedures, emergency care, and basic diagnostic services. The setting was chosen for its representative nature of sub-district hospitals in the region, which often act as critical points of care between primary health centers and higher-level district or tertiary hospitals. The study was conducted over a four-month period, from May 2023 to August 2023, allowing for the collection of sufficient data to identify trends and patterns in referral rates.

Data collection involved both qualitative and quantitative methods. Qualitative data were gathered through brainstorming sessions conducted with healthcare providers (such as doctors and nurses), administrative staff, and patients who had been referred to higher-level facilities. These sessions were designed to elicit in-depth insights into the reasons behind the referrals, perceived barriers within the hospital, and potential areas for improvement. The fishbone diagram, a tool commonly used in RCA, was employed to systematically capture and categorize these insights, enabling a clear visualization of the underlying issues contributing to increased referral rates.

On the quantitative side, referral data were meticulously collected from the hospital's records for two distinct periods: from May-June 2023 (prior to the implementation of any interventions) to July-August 2023 (after the implementation of a Plan-Do-Study-Act (PDSA) cycle aimed at addressing the identified issues). This data provided a quantitative measure of the extent and trends in referrals, allowing for a before-and-after comparison to assess the impact of the interventions.

The RCA process involved a detailed analysis of the collected data using the fishbone (Ishikawa) diagram. This diagram was used to categorize the causal factors contributing to increased referral rates into key areas such as resource limitations, staffing issues, clinical training gaps, infrastructure constraints, and patient-related factors. Each of these categories was explored in depth to identify specific issues that could be addressed to reduce referral rates. For instance, resource limitations might include the lack of essential medical equipment or insufficient diagnostic capabilities, while staffing issues could involve inadequate numbers of trained personnel or a lack of specialist doctors.

The data analysis also involved the use of Pareto and bar charts to present the quantitative referral data. The Pareto chart helped to identify the most frequent reasons for referrals, allowing the hospital to focus on the conditions that accounted for the majority of referrals. The bar chart was used to compare referral rates before and after the PDSA cycle, providing a visual representation of the effectiveness of the interventions.

## Results

**Figure 1:**
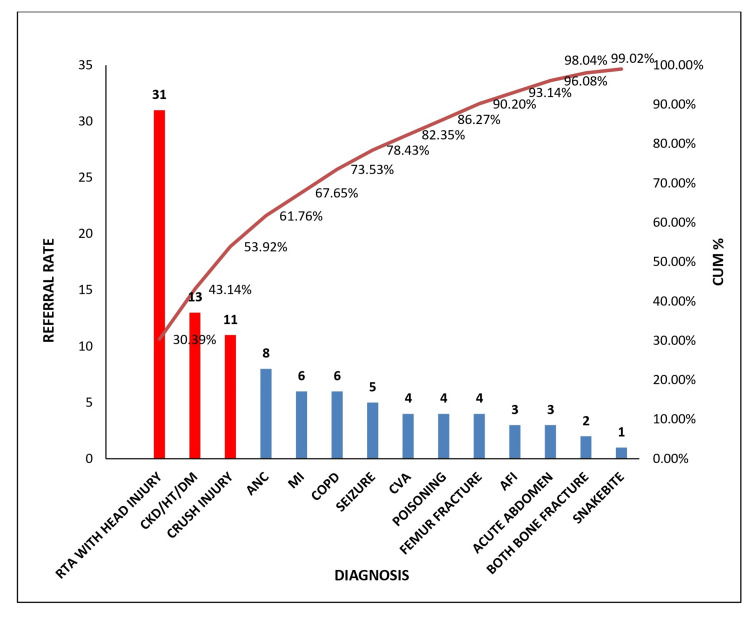
Referral rate for the month of May-June 2023 RTA: Road traffic accident; CKD/HT/DM: Chronic kidney disease/hypertension/diabetes mellitus; ANC: Antenatal case; MI: Myocardial infarction; COPD: Chronic obstructive pulmonary disease; CVA: Cerebrovascular accident; AFI: Acute febrile illness

Figure 1 shows a Pareto chart analysis of referrals from sub-district hospitals from May to June 2023, revealing that the most frequent referrals are road traffic accidents (RTAs) with head injury (31 cases, or 30.39%), chronic kidney disease (CKD)/hypertension (HT)/diabetes mellitus (DM) (13 cases, or 12.75%), and crush injury (11 cases, or 10.78%), which together account for 53.92% of all referrals. Other notable diagnoses include antenatal case (ANC) (eight cases, or 7.84%), myocardial infarction (MI) (six cases, or 5.88%), chronic obstructive pulmonary disease (COPD) (six cases, or 5.88%), and seizures (five cases, or 4.90%). The cumulative percentage curve shows that the top eight diagnoses collectively represent 78.43% of the total referrals, indicating that a focused effort on managing these high-frequency conditions could significantly reduce referral rates, thereby enhancing patient care and optimizing resource utilization within the sub-district hospital.

**Figure 2:**
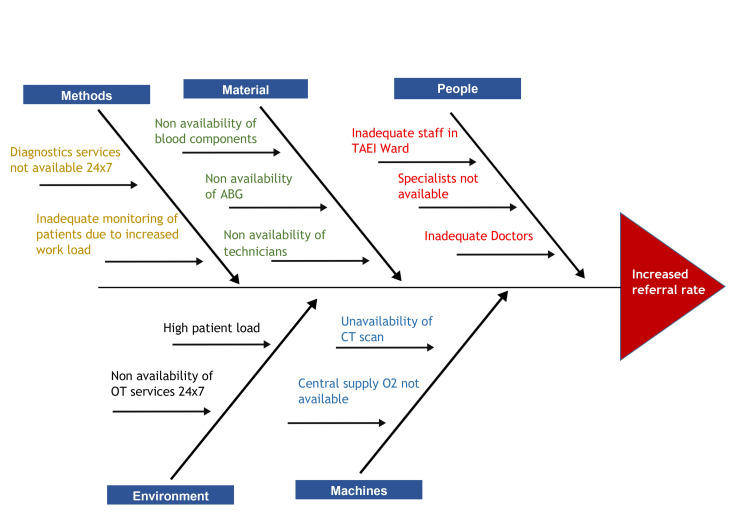
Fishbone diagram illustrating the root cause analysis of increased referral rate TAEI: Tamil Nadu Accident and Emergency Care Initiative; ABG: Arterial blood gas; OT: Operation theater; CT: Computed tomography

Figure 2 is a cause-and-effect diagram, also known as a fishbone or Ishikawa diagram, illustrating the root causes of the increased referral rate in the healthcare setting. The causes are categorized into five main groups: Methods, Material, People, Environment, and Machines. In the Methods category, the limited availability of diagnostic services 24x7 and the inadequate monitoring of patients due to increased workload are highlighted as key issues leading to more referrals. Under Material, the non-availability of blood components, arterial blood gas (ABG) analysis, and skilled technicians are significant factors. The People category points to inadequate staffing in the Tamil Nadu Accident and Emergency Care Initiative (TAEI) ward, the unavailability of specialists, and a general shortage of doctors as major contributors. Environmental factors include a high patient load and the non-availability of operating theater (OT) services around the clock, both of which exacerbate the referral problem. Finally, the Machines category highlights the unavailability of CT scans and the central supply of oxygen as critical issues leading to increased referrals. Each of these factors interlinks and collectively contributes to the higher referral rates, as depicted by the arrows converging on the central problem in the diagram.

**Figure 3:**
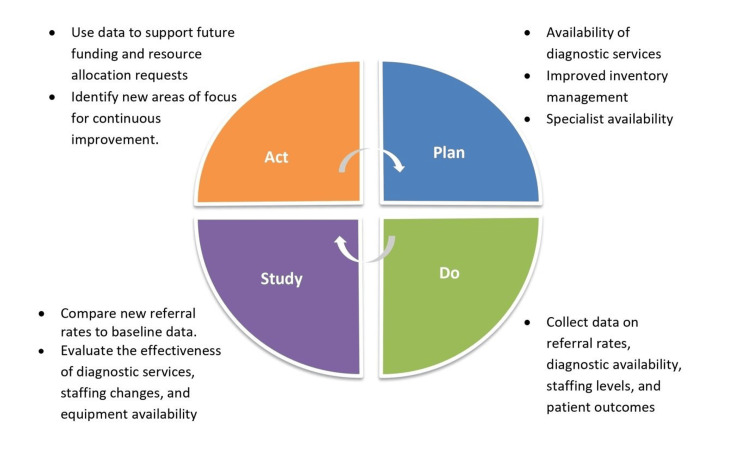
Plan-Do-Study-Act (PDSA) cycle to reduce the unwanted referral

To reduce the increased referral rates in our healthcare setting, we have initiated a comprehensive improvement plan structured around the PDSA cycle (Figure 3). First, we have enhanced the availability of diagnostic services by revising staffing schedules. Second, measures have been taken to ensure the continuous availability of essential materials, such as blood components and ABG analysis kits, through improved inventory management and backup supply arrangements. Third, addressing staffing shortages in critical areas like the TAEI ward and specialist availability has been prioritized through recruitment assessments and training programs. Additionally, efforts to manage high patient loads and optimize OT services have begun, focusing on streamlining patient flow and exploring extended service hours. These initiatives systematically tackle the root causes identified in our cause-and-effect analysis, fostering a more efficient healthcare environment with reduced referral rates over time.

**Figure 4:**
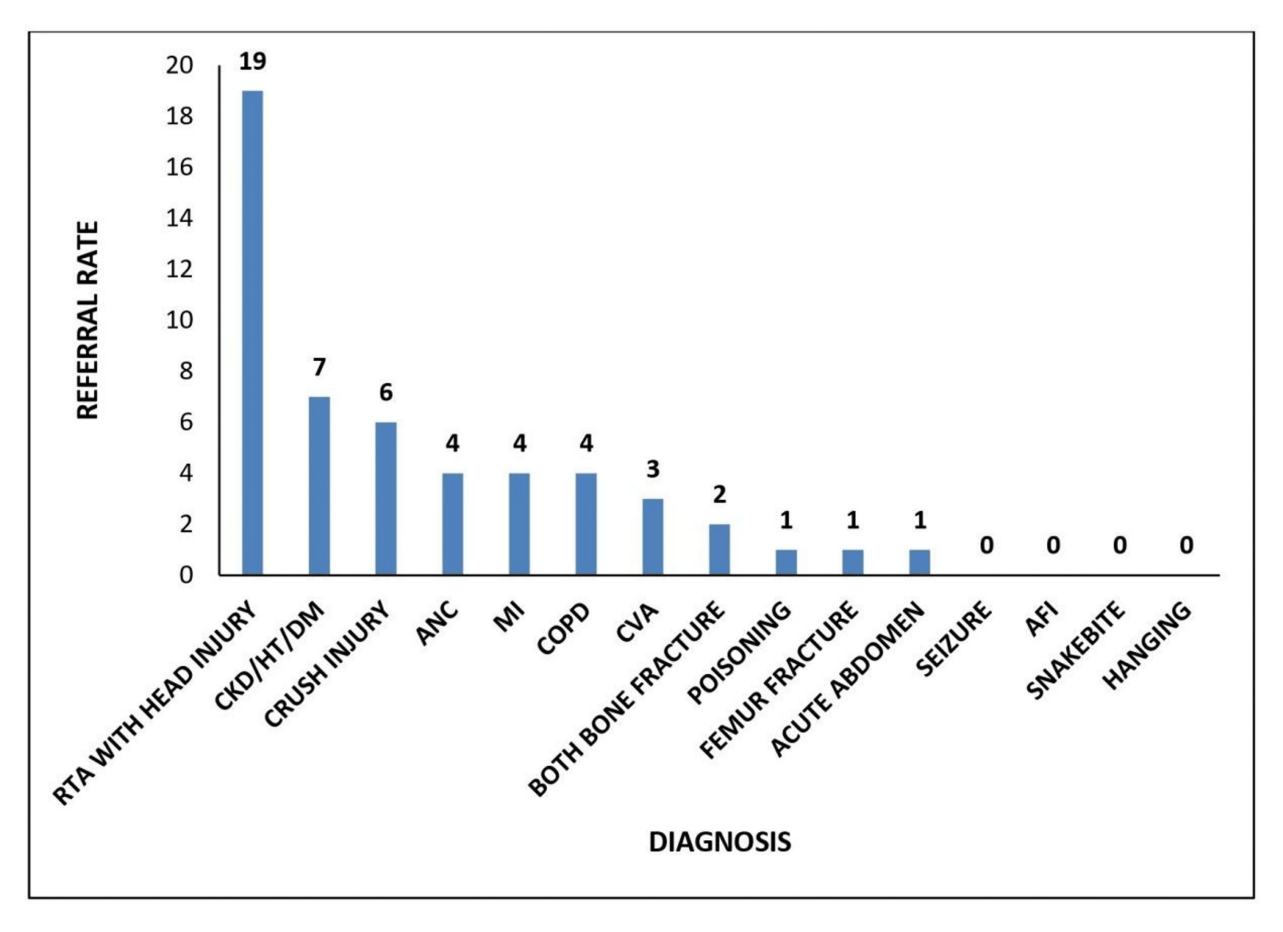
Referral rate for the month of July-August 2023 RTA: Road traffic accident; CKD/HT/DM: Chronic kidney disease/hypertension/diabetes mellitus; ANC: Antenatal case; MI: Myocardial infarction; COPD: Chronic obstructive pulmonary disease; CVA: Cerebrovascular accident; AFI: Acute febrile illness

The bar chart (Figure 4) shows referral data post-PDSA cycle, with RTAs with head injury as the most frequent referral at 19 cases, which is lower than the 31 cases reported in the Pareto analysis for May-June 2023, suggesting a reduction rate of approximately 38.7%. Similarly, referrals for CKD/HT/DM decreased from 13 cases to 7, a reduction rate of 46.2%, and crush injury referrals dropped from 11 to 6, a 45.5% decrease. As a result, there is a significant decrease in the referral rate, from 101 cases referred in May-June 2023 to 52 referral cases in July-August 2023. This overall reduction in referrals, reflected in the chart, suggests an effective PDSA cycle at the sub-district hospital.

Healthcare for patients at the sub-district hospital has significantly improved by addressing the root causes of the increased referral rate, identified through a comprehensive RCA. By tackling these underlying issues, the hospital has successfully reduced the number of referrals, enabling more patients to receive the necessary treatment locally. This not only enhances the quality of care at the sub-district level but also alleviates the patient burden on the district headquarters hospital, ensuring a more efficient and effective healthcare system overall.

## Discussion

The quality of emergency and trauma care in low- and middle-income countries, like India, is often understudied, and one study found that the mortality recorded in emergency departments is many times higher than that generally reported in high-income countries, pointing to gaps in the quality and appropriateness of services being provided in these emergency departments [4]. Quality should not be reserved for an elite few or viewed as a distant aspiration; it must be embedded in the very core of all health systems. Additionally, the human right to health loses its significance without high-quality care, as health systems cannot enhance health outcomes without it [5].

High-quality care encompasses a comprehensive approach that includes thorough assessments, identification of asymptomatic and co-existing conditions, precise diagnosis, timely and appropriate treatment, referrals for hospital care and surgery when necessary, and ongoing monitoring to adjust the treatment plan as needed [6]. District and tertiary healthcare facilities with advanced diagnostic and treatment options are often crowded with stable patients who, in turn, could have been treated in hospitals at sub-district levels. On the contrary, many primary healthcare centers and hospitals at sub-district levels deal with cases beyond their level of care, with a weak referral chain system [7,8].

Hence, the RCA, as part of our quality improvement (QI) initiative, which aimed at understanding the rise in referral rates at a sub-district hospital in Tamil Nadu, identified a range of systemic challenges and operational deficiencies. These issues have been impacting the quality of patient care and overall outcomes.

The lack of resources in the emergency department played a crucial role in increased referral rates. Firstly, the present study identified deficiencies in materials and types of equipment, such as the non-availability of CT scans, blood components, and ABG analysis, which were most vital in managing RTAs with head injury and crush injury, the major cases referred from the emergency room (ER). With proper and efficient inventory control and the installation of CT scans at sub-district hospitals, the number of RTA case referrals has been reduced by nearly 50%. This has also been evidenced by a study conducted by Shah et al. in Bangladesh [9] and Patil et al. in Maharashtra [10].

Secondly, our RCA showed inadequate recruitment of staff and a lack of proper training in emergency trauma care management. Job analysis and the recruitment of adequate specialists at the sub-district level had been done, which helped in managing cases and thus reducing the workload. Periodic work sampling measurement, emergency department organization behavior analysis, and capacity building can decrease the need for referrals. When referrals are necessary, improved emergency care can stabilize the patient’s condition, which, in turn, boosts the chances of a favorable outcome on reaching a tertiary healthcare facility [8]. Also, the employment of primary healthcare professionals/preventive healthcare professionals at ER triage has shown good results in patient care and management in the emergency department, as evidenced by studies conducted in China, Canada, and Oman [11-14].

According to the Sustainable Development Goals (SDGs) 2030, there are about 89 health-related indicators, among which 28 are quality-related indicators. Now that we are living in the SDG era, with the shift to non-communicable diseases and injuries [15], health emergencies are also on the rise. Hence, a rapid, sturdy, and stringent healthcare system that is adequately staffed and equipped with a fully functioning emergency department is vital in the pathway to achieving the ambitious SDG targets and thereby contributing to the better health and well-being of the nation [16,17]. The limitation of this study was that the findings might be highly specific to the sub-district hospital in Tamil Nadu and may not be generalizable to other settings or regions with different healthcare systems or patient populations.

## Conclusions

The RCA conducted on the increased referral rates from the sub-district hospital in Tamil Nadu has highlighted significant systemic and operational deficiencies that impact patient care and resource utilization. Through a detailed investigation using the fishbone diagram and PDSA cycle, we identified key areas, such as resource limitations, staffing issues, and infrastructure constraints, that contribute to unnecessary referrals. Implementing targeted improvements, including enhanced diagnostic services, better resource management, and improved staffing, has led to a notable reduction in referral rates, demonstrating the effectiveness of the QI initiatives. These findings underscore the importance of strengthening sub-district hospitals to ensure they can manage more cases locally, thereby reducing the burden on higher-level facilities and improving overall healthcare delivery. This approach aligns with global health goals by promoting high-quality care at all levels of the healthcare system.
